# Dietary melatonin supplementation mitigates the negative effects of artificial light at night in the Pacific field cricket, *Teleogryllus oceanicus*

**DOI:** 10.1098/rspb.2025.2072

**Published:** 2025-11-26

**Authors:** Nicola-Anne Jade Rutkowski, Theresa Melanie Jones, Kathryn B. McNamara

**Affiliations:** ^1^ School of Biosciences, The University of Melbourne, Melbourne, Victoria 3010, Australia

**Keywords:** light pollution, ALAN, development, melatonin, fitness

## Abstract

Artificial light at night (ALAN) is linked to negative behavioural and physiological consequences in animals. A potential mechanism for these adverse effects is artificial light at night’s inhibition of melatonin synthesis, a zeitgeber for cellular processes and a powerful antioxidant. Accordingly, melatonin supplementation can ameliorate artificial light at night-linked pathologies. Most studies expose animals to artificial light at night across their whole lifespan or a single life stage, but many nocturnal species experience variable exposure across heterogeneously lit landscapes. We investigated the effects of artificial light at night during both early- and late-juvenile development on adult reproduction in the Pacific field cricket *Teleogryllus oceanicus*, and whether dietary melatonin supplementation could mitigate these effects. We found life-stage-specific developmental effects of artificial light at night. Lifelong artificial light at night exposure accelerated juvenile development, yet did not affect late-juvenile development, total development time or adult body mass. Moreover, we confirm the potential for melatonin supplementation to rescue male sperm viability and daily egg production by females. The degree of ‘rescue’ was sex, and potentially age specific, which may be explained by the differential effect of artificial light at night on early-juvenile development. Critically, this effect would have been masked had we not partitioned this early life stage, underscoring the importance of considering life-stage-specific artificial light at night exposure when evaluating ecological and physiological consequences.

## 1. Introduction

Globally, natural night-time darkness is rapidly disappearing [1], creating widespread negative physiological and behavioural effects for animals [2–4]. Compelling evidence links the presence of artificial light at night (ALAN) to shifts in life-history traits such as development and growth [5–7], fecundity and survival [8–10], modifications to reproductive behaviours [11–13] and circadian timing [14,15]. To date, the majority of studies investigating the ecological effects of ALAN have experimentally manipulated intensity [5,9,16], spectra [17,18] or duration over a 24 h period [19,20]. Moreover, it is clear that both long-term (chronic exposure, typically over the lifetime) [13,21,22] and short-term (acute exposure, at either the adult and/or juvenile phase) [23–26] exposure can have negative biological effects. However, to our knowledge, no study has explored ALAN’s impact on partitioned early- and late-juvenile stages of development. This is an important knowledge gap in hemimetabolous invertebrates as these two stages represent different phases of resource allocation: early-juvenile stages invest heavily in somatic growth [27,28]; late-juvenile stages must trade off somatic investment against investment into reproductive growth [29,30].

Regardless of the life stage, a potential mechanism underpinning the deleterious effects of ALAN is its interaction with melatonin, a circadian driver [31] and powerful antioxidant [32]. Melatonin is a zeitgeber of all cellular processes [33] synchronizing an organism’s internal circadian rhythms to that of the Earth’s light cycles [34]. Consequently, melatonin is involved in, and often governs, the timing of biological processes, from the daily to the seasonal [31,35]. Indeed, the circadian synthesis of melatonin is known to play a clear role in vertebrate photoperiodism [36], although this relationship is less established in invertebrates [37]. Moreover, melatonin protects organisms from oxidative stress by interacting with reactive oxygen species (ROS) [38,39]. This has demonstrable carry-over effects for survival [40,41], fertility [42], sperm viability [43,44] and spermatogenesis for both vertebrates and invertebrates [45,46]. The melatonin pathway and its synthesis are photosensitive (particularly to short wavelength blue light), typically resulting in a peak in production during the night and naturally lower levels during the day [3]. This is problematic as ALAN is often rich in blue light, mimicking natural light, thus its presence can suppress melatonin synthesis, resulting in reduced concentrations of circulating melatonin during periods when it should be naturally at its highest [47–51]. This may drive mismatches in circadian processes and function, as well as having inevitable consequences for an animal’s antioxidant levels.

The impact of ALAN exposure and circulating melatonin concentrations on physiology and behaviour has produced equivocal patterns. While most studies find that ALAN reduces concentrations of circulating melatonin [47,48,50,52,53], this is not always associated with consistent changes in behaviour or physiology. For example, in Great tits, *Parus major*, changes in activity patterns are directly linked to ALAN-related reductions in melatonin [47]. Similarly, in rats, ALAN disrupts circadian rhythms in the cardiovascular system and suppresses circulating concentrations of melatonin [53]. However, in roach, *Rutilus rutilus,* cortisol levels were unaffected by ALAN despite the reduction in circulating levels of melatonin [52]. Studies where circulating melatonin concentrations are directly manipulated, via dietary supplementation [54–56] or transdermal patches [57,58], offer an alternative approach, providing direct evidence of the causational relationship between ALAN-induced melatonin shifts and associated physiological and behavioural trait responses. Accordingly, experimental supplementation with dietary melatonin during adult exposure to ALAN assists recovery of immune function in crickets and quail [54,59], reduces oxidative stress in rats [55,60] and diminishes ALAN-induced stress responses in budgerigars [61]. However, the recovery effect of melatonin has only been explored over a narrow range of traits. Given melatonin’s role as a time keeper of all cellular processes, it is likely to influence other temporally governed traits that are disrupted by ALAN, such as growth and development [5,7,62], sperm viability [63–65] and fecundity [9,10]. Moreover, whether melatonin supplementation is effective in mitigating the effects of variable ALAN exposure over different life-history stages (defined here as acute or chronic ALAN exposure—see below) has, to our knowledge, never been considered.

The Pacific field cricket, *Teleogryllus oceanicus*, is an ideal species to explore the potential of melatonin to rescue fitness following various periods of ALAN exposure. A primarily nocturnal species, *T. oceanicus,* inhabits rural (no light at night) and urban (brightly lit) landscapes in the western and northern coasts of Australia [66]. During its lifetime, *T. oceanicus* undergoes high periods of movement and dispersal, particularly in the final juvenile instar and adult stages [67], making testing both chronic and acute ALAN exposure ecologically relevant for this species. The life-history impacts of ALAN have been explored in the congeneric species, *T. commodus*, where exposure to ALAN elongated juvenile development time [5], increased body size [5], decreased immune function [21,49], altered courtship and mating behaviour [13], and influenced movement [68]. Similarly, the impact of ALAN on circadian behaviours and gene expression has recently been studied in the two-spotted cricket, *Gryllus bimaculatus* [15,69]. However, the relative importance of ALAN exposure during the early- and late-juvenile phase is untested. Here, we exposed *T. oceanicus* individuals to ALAN*,* from either the first instar onwards (defined as a chronic treatment) or penultimate juvenile instar onwards (defined as an acute treatment). Once they had reached the penultimate juvenile instar, surviving crickets were housed individually (see below), and half were supplemented with dietary melatonin to assess the efficacy of exogenous melatonin to mitigate the impact of ALAN on key life-history traits. We predicted that exposure to ALAN would affect juvenile growth and adult body mass, as observed in the congeneric species *T*. commodus [5,21]. We further predicted that male sperm viability and female fecundity would be reduced in ALAN-exposed individuals, as ALAN compromises melatonin synthesis, escalating oxidative stress [70]. We expected that ALAN-related effects would be ‘exposure-duration-dependent’ [47] and thus be most pronounced for individuals chronically exposed. Finally, we predicted we would see a rescue effect of melatonin supplementation on some, if not all, traits measured via melatonin’s ability to react with, and reduce, ROS [54,55,59–61].

## 2. Methods

### (a) Stock population

Experimental crickets were obtained from a large out-bred stock laboratory population (>1000 individuals) *of T. oceanicus* originally collected from Carnarvon, Western Australia (24.8839° S, 113.6571° E) and supplemented every 2 years with new field-caught crickets. Both stock and experimental individuals were reared in large containers (25 × 14 × 17 cm) containing egg cartons for shelter, *ad libitum* water and dried cat food (Smitten kitten food, Bella Vista, New South Wales, Australia) and maintained under constant conditions (25°C; 12 h light : 12 h dark). The position of stock and experimental animal containers was rotated weekly across multiple shelves.

### (b) Manipulation of artificial light at night and melatonin exposure

To investigate the ability of melatonin supplementation to rescue individuals from the negative effects of ALAN exposure, we simultaneously manipulated the presence and duration of ALAN and melatonin supplementation. Experimental crickets were initially exposed to one of three ALAN treatments: (i) *dark treatment crickets* were exposed to night-time light levels that approximated the light emitted by a new moon (0.001 lux (lx)) for the duration of the experiment, (ii) *ALAN-acute treatment crickets* were exposed to night-time light levels that approximated the light emitted by a new moon (0.001 lx) until their penultimate juvenile moult, after which time night-time light levels were increased to approximate a streetlight (10 lx) and (iii) *ALAN-chronic treatment crickets* were exposed to night-time light levels that approximated a streetlight (10 lx) for the duration of the experiment. All individuals, regardless of treatment, were maintained on an identical 12 h daylight : 12 h nightlight treatment lighting schedule (daytime light intensities = 2000 lx). Lighting was created using standard cool-white (having a higher proportion of blue light) 6000 Kelvin light emitting diode light strips (Jaycar, ZD0552, Australia), and the different day and night lux levels were manipulated using a lighting controller (STICK-DE3, Nicolaudie Architectural). Calibration of lux levels was done weekly, by placing a light meter (ExTech Instruments EasyView 33) at the ground level of the containers.

Experimental individuals were group-reared in large, clear containers (25 × 14 × 17 cm; *n* = 20 replicate boxes) at an initial density of 100 individuals until the pre-penultimate instar, when they were transferred to individual plastic containers (7 × 7 × 5 cm) with *ad libitum* dried cat food (as above) and water, with a single egg carton for shelter. Following their penultimate eclosion, all individually housed crickets were provided with drinking water that contained either a solution of 50 μg ml^-1^ of melatonin or a 0 μg ml^-1^ control (following [54,56], see electronic supplementary materials for more details). Melatonin and control solutions were provided in 2 ml blacked-out vials (to reduce the impact of light on melatonin degradation), plugged with solution-soaked cotton wool. Vials were changed twice weekly for the duration of the experiment. Upon adult eclosion, individuals were assigned to assays to determine the impact of ALAN and melatonin exposure on life-history traits (development, morphology and adult fitness). All assays were conducted blind to experimental treatment.

### (c) Juvenile development, adult morphology and survival

To assess early-juvenile survival, we counted the mean proportion of individuals surviving to the penultimate instar from each replicate rearing box (*likelihood that juvenile survived to the penultimate stage*). All individuals were monitored daily until adult eclosion (*time from egg to penultimate, time from penultimate to adulthood*). We recorded the sex and body mass (to the nearest mg) of each adult 7 days post-adult eclosion and monitored all adults daily until death or for 19 days post-eclosion after which time they were frozen (−80°C).

### (d) Reproductive investment

To assess the impact of ALAN duration and melatonin supplementation on female fitness, we measured the number of eggs produced by twice-mated females. Here, 10-day-old virgin experimental females (mean age ± s.e. = 10.53 ± 0.90) were transferred to a container (7 × 7 × 5 cm), with a virgin 10 day old stock male (mean age ± s.e. = 10.45 ± 0.05) and left for 30 min to mate. If the female did not mate within 30 min, the male was substituted with a second virgin male and the pair again left for 30 min. After mating, the stock male was removed, and the mated female left for 2 h prior to being presented with a novel virgin male (mating conditions as above). If a female mated once only after the presentation of two males, she was returned to her container, left for 24 h, and then the above process was repeated. Any female that failed to mate twice in the allocated 48 h period was not included in the fecundity data but was checked daily for survival until day 19 after which time they were frozen at −80°C. Twice-mated experimental females were transferred to a new container (7 × 7 × 5 cm) containing water (containing their respective melatonin dosage), *ad libitum* cat chow and a sand pad (4 × 1.5 cm Petri dish with damp sand) and returned to their adult light treatment for oviposition. Sand pads were replaced every 3 days for a total of 9 days after which time females were frozen at −80°C. Eggs were rinsed from the sand and the total number of eggs laid over each 3 day period was counted.

To explore variation in male fitness across the ALAN and melatonin treatment groups, we assessed sperm viability (a standard predictor of male competitive fertilization success in this species [71]) at day 10 (mean age ± s.e. = 10.56 ± 0.07) and day 14 (mean age ± s.e. = 14.24 ± 0.06) post-adult male eclosion. These two time periods were chosen as sperm viability naturally declines over this range [72]. To ensure assayed spermatophores were of comparable age, 4 h before assessment, the male’s genital pouch was inspected for the presence of a spermatophore which, if present, was removed with fine forceps (following Kerr *et al.* [73]). Four hours is typically sufficient time for a new spermatophore to be produced in this species [74]. At the start of the trial, the fresh (<4 h old) spermatophore was removed and sperm viability assessed using the Live:Dead Sperm Viability Kit (Molecular Probes, Eugene, OR, USA) (following McNamara [75]; see Supplementary Material for a full description).

### (e) Data analysis

Data were analysed using R (v.2023.12.1+402) [76,77]. Variables were assessed for normality prior to analysis and transformed to maximize the normality of the model residuals (the exponent used was recorded for each analysis, included within the analysis table). For all models (unless otherwise stated), ALAN treatment, melatonin dose, sex (and their interaction) and body weight were entered as fixed effects. Only the interaction between ALAN × melatonin was retained in final models regardless of significance; other interactions and fixed effects with *p* > 0.1 were removed [78]. The origin box of each individual was included as a random effect (‘origin box’) in all models. *Post hoc* pairwise comparisons were conducted, where stated, using the Holm correction [79]. For planned comparisons of interaction effects, if one of the two fixed effects was non-significant, we held that effect constant. Sample sizes for all models are included in Supplementary Material (electronic supplementary material, table S1).

We used linear mixed-effect models to analyse the time from egg to penultimate eclosion, time from penultimate to adult eclosion and body weight. For egg to penultimate eclosion, we did not include melatonin dosage as a fixed effect, as it had not been administered at this stage. For the time from penultimate to adult eclosion, the time from egg to penultimate eclosion was initially included as a fixed effect but was dropped as *p* = 0.98. For males, we also ran linear mixed-effect models to analyse sperm viability on days 10 and 14. Prior to analysis, the proportion of viable sperm was arcsine transformed. Three data points were removed as sperm viability could not be assessed due to poor image quality (day 10 = 1 male; day 14 = 2 males). For analysis of sperm viability at day 14, whether males produced a spermatophore on day 10 was included as a fixed effect. Where significant effects were found, we conducted planned comparisons. Comparisons were only made within light treatments to assess differences between melatonin levels (0 ml ml^−1^ versus 50 ml ml^−1^). Six of the 71 females (who laid more than 10 eggs) escaped prior to the last egg collection; thus, we used the number of eggs laid per day as our metric of female fecundity.

We used generalized linear models with a binomial error distribution to compare the likelihood of surviving to penultimate eclosion for ALAN and Dark treatments (acute individuals were not yet exposed to ALAN and thus included in the Dark treatment), the likelihood of a female mating twice and the likelihood of a male producing a spermatophore on days 10 and 14 (see electronic supplementary material, table S2). Melatonin was not included as a fixed effect in early-juvenile models, as it had not been administered at that time point. Whether males produced a spermatophore on day 10 was included as a fixed effect for day 14.

## 3. Results

### (a) Early survival, juvenile development and body mass

**Figure 1.**
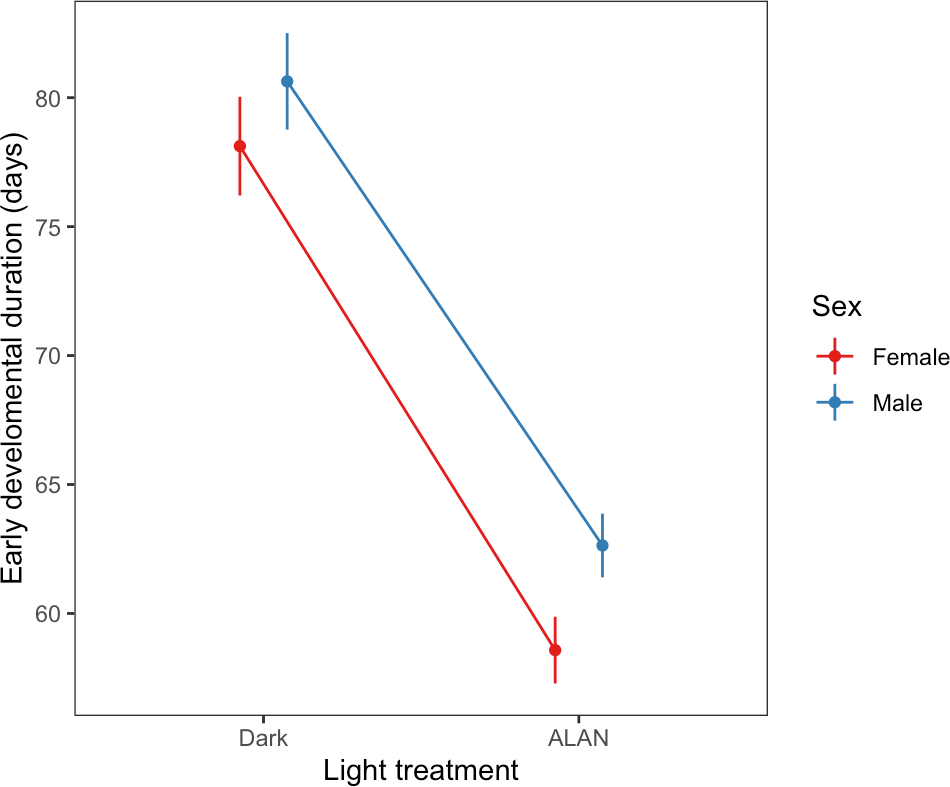
The effect of light treatment on early developmental duration in days (egg to penultimate instar) in males and females. Points represent mean durations; errors are standard errors about the mean.

The time from egg to the penultimate instar (our metric of early-juvenile development and prior to the administration of melatonin) was approximately 18 days (25%) shorter for crickets reared under ALAN compared twiththose reared under darkness (mean ± s.e. ALAN = 60.60 ± 0.91; Dark = 79.3 ± 1.34, table 1; figure 1a). In contrast, exposure to ALAN did not affect the likelihood that a juvenile cricket survived to the penultimate instar (F_1,18_ = 0.15, *p* = 0.71), the time from penultimate instar to adult (our metric of late-juvenile development and when melatonin supplementation treatment commenced) or the total time from egg to adult (total juvenile development time; table 1). However, late- and total-juvenile development times were longer for males (table 1) and tended to be shorter for melatonin-supplemented individuals (table 1). Despite the reduced time for development, adult females were heavier than males (mean ± s.e. female body mass = 0.73 ± 0.008; male body mass = 0.71 ± 0.006, table 1), but body mass was unrelated to either light or melatonin treatment.

**Table 1.** The effect of light treatment and melatonin supplementation on juvenile development, body mass, and female and male reproductive parameters. Values in bold have *p*-values < 0.05. Superscript letters indicate exponent transformations to meet normality assumptions: ^a^ ^0.04; ^b^ ^1.6; ^c^ ^0.24; ^d^ ^3.52; ^e^ ^2.92. Sperm viability measures were also arcsine transformed prior to analysis.

	light treatment	melatonin	light treatment x melatonin	sex	weight	day 10 spermatophore?
sexes combined						
egg-penultimate duration (days)^a^	***χ*^2^_1_ = 4.97**			*χ*^2^_1_ = 2.84		
	***p* = 0.03**			*p* = 0.09		
penultimate-adult duration (days)^a^	*χ* ^2^_2_ = 2.45	*χ*^2^_1_ = 2.97	*χ* ^2^_2_ = 0.68	***χ*^2^_1_ = 42.42**		
	*p* = 0.29	*p* = 0.08	*p* = 0.71	***p* < 0.01**		
egg-adulthood (days) ^a^	*χ* ^2^_2_ = 5.09	*χ*^2^_1_ = 0.66	*χ* ^2^_2_ = 2.93	***χ*^2^_1_ = 8.81**		
	*p* = 0.08	*p* = 0.42	*p* = 0.23	***p* = 0.003**		
body mass (mg)^b^	*χ* ^2^_2_ = 0.89	*χ* ^2^_1_ = 0.74	*χ* ^2^_2_ = 3.11	***χ*^2^_1_ = 9.67**		
	*p* = 0.64	*p* = 0.39	*p* = 0.21	***p* < 0.01**		

females						
likelihood of two matings	*χ* ^2^_2_ = 0.36	*χ*^2^_1_ = 3.44	*χ* ^2^_2_ = 2.08		***χ*^2^_1_ = 4.48**	
	*p* = 0.84	*p* = 0.06	*p* = 0.35		***p* = 0.03**	
number of eggs laid^c^	*χ* ^2^_2_ = 0.28	***χ*^2^_1_ = 10.17**	***χ* ^2^_2_ = 7.82**			
	*p* = 0.87	***p* < 0.01**	***p* = 0.02**			

males						
sperm viability (day 10)^d^	*χ* ^2^_2_ = 1.45	*χ*^2^_1_ = 0.0004	*χ* ^2^_2_ = 2.98			
	*p* = 0.49	*p* = 0.99	*p* = 0.23			
sperm viability (day 14)^e^	*χ* ^2^_2_ = 0.42	***χ*^2^_1_ = 6.05**	***χ* ^2^_2_ = 7.18**		***χ*^2^_1_ = 4.43**	*χ*^2^_1_ = 1.77
	*p* = 0.81	***p* = 0.01**	***p* = 0.03**		***p* = 0.03**	*p* = 0.18

### (b) Reproductive investment

**Figure 2.**
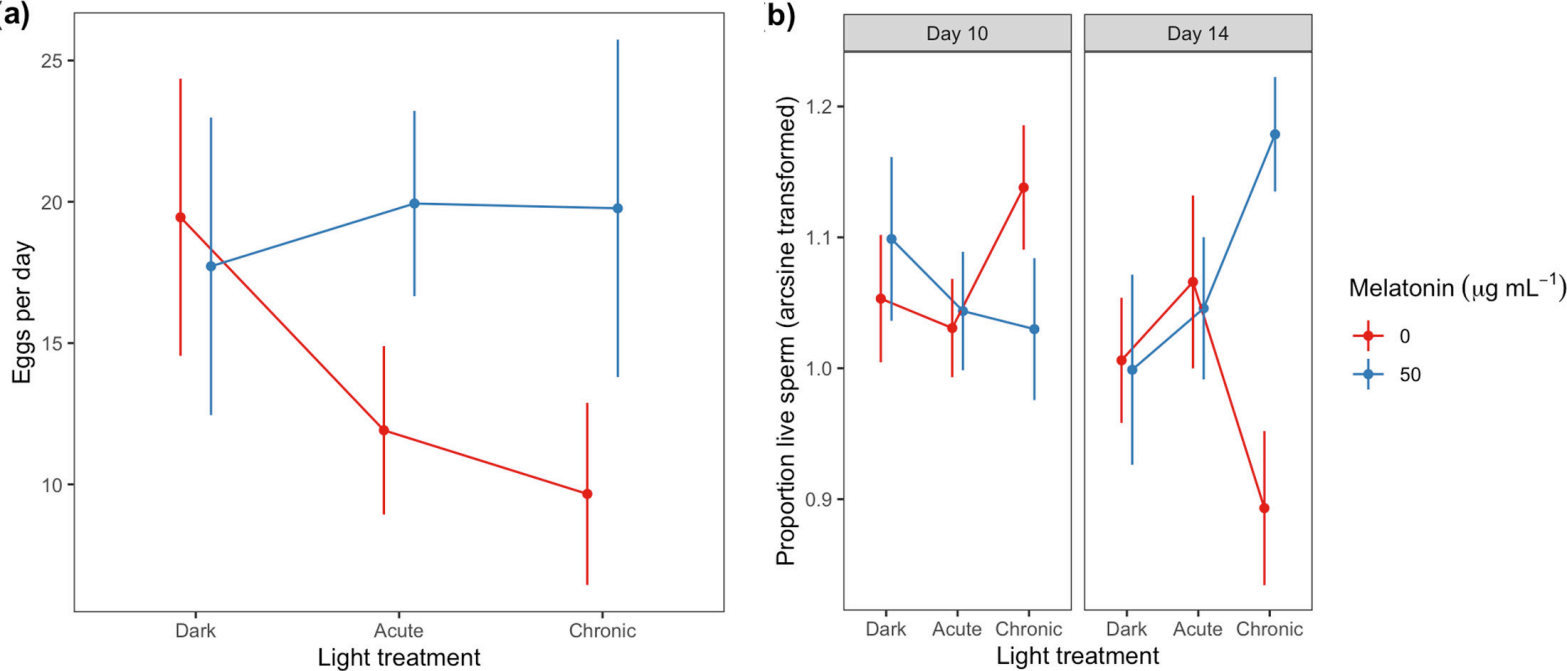
The effect of light treatment and melatonin supplementation on (a) female reproductive output (eggs per day) and (b) sperm viability (proportion of viable sperm). Pairwise comparisons adjusted using the Holm method identified significant differences in (a) between acute 0 µg ml^−1^ and 50 µg ml^−1^ (t = −2.30, d.f. = 70.3, *p* = 0.05) and chronic 0 µg ml^−1^ and 50 µg ml^−1^ (t = −3.51, d.f. = 63.7, *p* = 0.002) and in (b) between chronic 0 µg ml^−1^ and 50 µg ml^−1^ (t = −3.54, d.f. = 70.5, *p* = 0.002). Notation as for figure 1.

*Females*—the likelihood of a female mating twice was negatively related to her body mass (*n* = 93/123 females; β (s.e.) = −6.47 (3.06)) and tended to be lower for melatonin-supplemented individuals (% successfully twice-mated females = 83% 0 µg ml^−1^ melatonin; 71%; 50 µg ml^−1^ melatonin females; table 1). The average number of eggs laid per day was comparable across the three lighting treatments (table 1, figure 2a). However, this is driven by the significant rescue effect of melatonin supplementation for acute and chronic (but not Dark) treatment females (table 1, figure 2a). *Post hoc* tests revealed that acute and chronic treatment females supplemented with 50 µg ml^−1^ melatonin laid significantly more eggs (67 and 105%, respectively) compared with chronic and acute females supplemented with 0 µg ml^−1^ melatonin (comparison between 0 and 50 µg ml^−1^ melatonin acute females: t = −2.30, d.f. = 70.3, *p* = 0.05; chronic females: t = −3.54, d.f. = 70.5, *p* = 0.002; table 1; figure 2a). In contrast, Dark treatment females produced a comparable number of eggs regardless of their melatonin treatment.

*Males*—the probability that a male produced a spermatophore on day 10 (94/122 males) or 14 (85/108 males) was unrelated to any variable measured (electronic supplementary material, table S2). However, sperm viability varied significantly with light and melatonin treatment for day 14 (but not day 10) males (table 1). For day 14 males, supplementation with dietary melatonin (50 µg ml^−1^) was linked to a 32% increase in sperm viability for individuals in the chronic treatment (t = −3.54, d.f. = 70.5, *p* = 0.002; table 1; figure 2b). Sperm viability was also positively related to the body weight of day 14 (but not day 10) males (table 1).

## 4. Discussion

Our data revealed three key findings related to the physiological effects of variation in exposure to ALAN and the potential for dietary melatonin supplementation to act as a rescue agent. First, exposure to ALAN had life-stage-specific developmental effects: its presence resulted in a 25% reduction in the time a nymph took to reach the penultimate instar. However, it did not strongly affect the time taken to complete the final juvenile moult, the overall development time or adult body size. Second, supplementation with dietary melatonin rescued some (albeit not all) ALAN-related reproductive declines in both males and females. Finally, we found sex-specific differences in developmental duration and adult weight that were unrelated to our two applied treatments. However, we suggest that differences in male and female development may explain the observed sex-specific differences in melatonin’s efficacy as a mitigator of light-related reproductive declines.

### (a) Life-stage-specific developmental effects of artificial light at night

ALAN is a well-documented developmental stressor, although the direction of its impact with respect to growth is inconsistent. Studies variously report increasing [5,80,81], decreasing [6,7,62,82,83] and no effect [9,84] of exposure to ALAN on the duration of juvenile growth. Here, we demonstrate that, regardless of the duration of exposure to light at night and despite marked differences in the early-juvenile phase, *T. oceanicus* juveniles complete their final moult in comparable (albeit sex-specific) timeframes. While we do not have definite evidence, it is highly likely that the final juvenile instar represents a period of increased and potentially compensatory feeding [85,86] and rapid weight gain [87]. The outcome of such a strategy is that, in the absence of a stage-specific experimental approach (such as the one employed here), these early life-stage impacts of ALAN would be effectively masked and would thus have been undetected. Whether this holds for other taxa, particularly those where no effect of ALAN is reported [9,84], remains to be investigated. This is not an insignificant knowledge gap because, as we suggest below, these early-juvenile developmental impacts can have substantial repercussions for adult reproduction and therefore fitness.

The impact of ALAN on *T. oceanicus* development and size provides an intriguing contrast when compared with its sister species, *T. commodus.* In *T. commodus*, ALAN prolongs juvenile development resulting in 15% larger adults under even very dim light at night (range of light treatments = 1, 10, 100 lx) [5]. It is possible to speculate that difference may reflect the two species’ evolutionary history and more specifically how they respond to variation in light. *Teleogryllus commodus* is a latitudinally widespread, seasonal breeder that responds to natural annual variation in day length [88]. Longer days (indicative of the onset of a resource plenty spring and potentially mimicked by the presence of ALAN) are suggested to promote prolonged juvenile development resulting in larger, more fecund adults [5]. In contrast, *T. oceanicus* occurs mainly in lower latitudes with relatively constant photoperiods [89] and is therefore less likely to use daily fluctuations in light as an abiotic cue for breeding [88,90]. Instead, for this species, ALAN may represent a significant environmental stressor that drives the rapid development of the more vulnerable early-juvenile stages [91]. Such shifts are likely mediated by melatonin, which has a regulatory role in circadian behaviour [34] and moulting [92,93] although our experimental design (which did not commence melatonin supplementation until the penultimate instar) precludes confirmation of this mechanism. Instead, it is more likely that the initial timing of supplementation would favour melatonin’s involvement in reproductive tissue rather than somatic growth [94].

### (b) Dietary melatonin as a reproductive rescue agent

The negative reproductive consequences of exposure to ALAN are well documented across taxa [7,12,63–65]. Similarly, the role for melatonin suppression as a driver of reproductive declines is frequently posited although the causal link is rarely demonstrated [54,95–97]. Here, we directly tested melatonin’s role using a supplementation approach and found improved reproductive performance for all ALAN-exposed females and for some (older, chronically exposed) males. Comparable data from melatonin supplementation studies on vertebrates report positive effects for egg laying [98,99], oocyte number and quality [42,100] and reductions in intra-follicular oxidative damage [101]. Moreover, in vertebrate males, melatonin occurs naturally in seminal fluid [95] and is used as a supplement to reduce ROS-induced cellular damage during sperm cryopreservation [102,103].

Sperm cells, in particular, are vulnerable to oxidative damage [70,104] which inevitably reduces male fertility [105–108]. Accordingly, in vertebrates age-related declines in the antioxidant capacity of seminal fluid [101,109–111] and concomitant increases in oxidative stress markers are reported [109,110]. Sperm viability in male *T. oceanicus* also naturally declines with age [72], but sperm competitiveness can be rescued with dietary antioxidant supplementation [104]. Following from this, it is likely that our older males (14 days) all had age-related elevations in oxidative stress, which were compounded for those exposed to a lifetime of ALAN, resulting in the observed reduction in sperm viability. We are unable to confirm whether oxidative stress and thus melatonin supplementation influenced the spermatozoa themselves or the seminal fluid that carries them. However, given the known importance of seminal fluid compounds in reducing the production of free radicals in vertebrates [112] and upregulation of antioxidant enzymes [113,114], it seems likely that it assisted age-related declines in seminal fluid, which were exacerbated under ALAN.

### (c) Sex-specific differences in light-related reproductive declines

Why significant shifts in sperm viability were apparent after chronic and not acute ALAN exposure remains unclear. We offer two mutually non-exclusive hypotheses. First, while the accelerated early larval development experienced by individuals exposed to chronic ALAN did not translate into significant differences in total juvenile development time or adult body size, it may have longer- term impacts on sperm viability. In *T. commodus,* individuals reared at high density (a social stressor) had accelerated juvenile development and males had reduced numbers of spermatozoa [115]. Although we did not assess sperm number, it is possible that the developmental acceleration associated with chronic ALAN exposure may have resulted in a comparable decline in sperm viability in *T. oceanicus*. However, the two stressors—ALAN and crowding—differ fundamentally in both ecological nature and physiological pathways. Second, spermatogenesis likely commences during early-juvenile development. In the closely related sister species, *T. commodus,* it starts at around 21 days of age and continues until a few days before the adult moult [115,116]. If this holds for *T. oceanicus*, which seems highly likely given the two species successfully hybridize when in sympatry [117], acute ALAN treatment males were only exposed to ALAN after the majority of spermatogenesis had been completed, reducing any ‘exposure-duration-dependent’ effects. This latter hypothesis may also explain why, for females, we see ALAN-related declines in the number of eggs laid regardless of the timing of first exposure.

Our data revealed important insights into the role of melatonin in rescuing reproductive output in females exposed to any ALAN during their lifetime. While the number of eggs laid per day was comparable for Dark females regardless of melatonin treatment (figure 2a), when supplemented with melatonin, acute and chronic females laid significantly more eggs (67% and 105%, respectively) compared with females who were not supplemented. Why we see such declines among both ALAN treatments likely relates to the timing of reproductive investment. In female insects, prominent changes occur in the ovaries after the final juvenile moult [30,118,119]. Indeed, for female two-spotted crickets, *Gryllus bimaculatus,* ovary weight increases from 20 mg to over 300 mg in the first 48 h of adulthood [119], and vitellogenesis reaches its highest rate between days 2 and 6 post-adult emergence [30]. Therefore, given chronic and acute females both experienced ALAN during the penultimate moult and adult egg development, any direct effects of ALAN on reproductive investment (in this case daily egg production) would be comparable.

Another explanation for the differing reproductive responses between the sexes could be sex-specific oxidative vulnerability. Sperm and egg cells exhibit different vulnerabilities to oxidative stress [120,121], with sperm cells more susceptible to damage [107,122]. However, our results conflict with this hypothesis, as only chronic males had reduced sperm viability, while both acute and chronic females had reduced egg laying. The reasons for such results remain unclear but warrant future research.

In conclusion, our study has revealed previously overlooked within-life-stage developmental effects of ALAN and provided further confirmation for the role of dietary melatonin as a potential mitigation strategy to counter its negative effects. Critically, our experiment suggests that, while exposure to ALAN during the early-juvenile stage has apparently limited knock-on effects for total juvenile development, it may have serious reproductive consequences (particularly for males) resulting in substantial reductions in fertility. However, we note that as our study does not directly measure melatonin levels, the mechanisms behind these consequences still remain unclear and should be the focus of future research. Nonetheless, for *T. oceanicus*, a species that breeds year-round, the expedited development and indeed some associated reproductive declines resulting from ALAN may not represent a significant stress to the system. However, for highly seasonal univoltine species, whose population viability relies on a short, highly concentrated reproductive season, ALAN may be disproportionately more serious.

## Data Availability

R code and data are available on Dryad [123]. Supplementary material is available online [124].
